# Immortalizing Cells for Human Consumption

**DOI:** 10.3390/ijms222111660

**Published:** 2021-10-28

**Authors:** Emily Soice, Jeremiah Johnston

**Affiliations:** 1 School of Science, Massachusetts Institute of Technology, 182 Memorial Drive, Cambridge, MA 02142, USA; ehsoice@mit.edu; 2 School of Humanities, Arts, and Social Sciences (SHASS), Massachusetts Institute of Technology, 182 Memorial Drive, Cambridge, MA 02142, USA; 3 New Harvest, 288 Norfolk Street, 4th Floor, Cambridge, MA 02139, USA

**Keywords:** cultured meat, cellular agriculture, immortalization, cell lines, genetic modification

## Abstract

The need to produce immortal, food-relevant cell lines is one of the most pressing challenges of cellular agriculture, the field which seeks to produce meat and other animal products via tissue engineering and synthetic biology. Immortal cell lines have a long and complicated story, from the first recognized immortal human cell lines taken from Henrietta Lacks, to today, where they are used to assay toxicity and produce therapeutics, to the future, where they could be used to create meat without harming an animal. Although work in immortal cell lines began more than 50 years ago, there are few existing cell lines made of species and cell types appropriate for cultured meat. Cells in cultured meat will be eaten by consumers; therefore, cultured meat cell lines will also require unique attributes not selected for in other cell line applications. Specifically, cultured meat cell lines will need to be approved as safe for consumption as food, proliferate and differentiate efficiently at industrial scales, and have desirable taste, texture, and nutrition characteristics for consumers. This paper defines what cell lines are needed, the existing methods to produce new cell lines and their limitations, and the unique considerations of cell lines used in cultured meat.

## 1. Introduction

**Figure 1:**
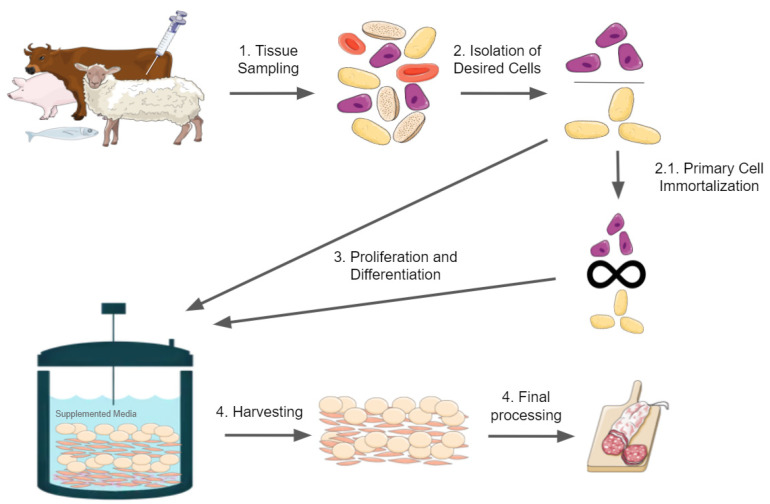
Brief overview of cultured meat production. The figure was constructed with illustrations taken from Servier Medical Art, licensed under a Creative Commons Attribution 3.0 Unported License.

Cellular agriculture is an emerging field that aims to produce animal products from cell cultures rather than whole animals. One of the anticipated products of cellular agriculture is cultured meat made in vitro from animal cell cultures, also known as cell-based meat, cultivated meat, in vitro meat, clean meat, artificial meat, or lab-grown meat. Multiple names exist due to a lack of consensus on a term that is both informative to consumers and accurate by technical and regulatory standards [1,2,3,4]. Global demand for meat is expected to increase by 1.3% per year on average from 2005 to 2050 as the global population and per capita consumption of meat grows [5]. The development of cultured meat products will require overcoming major technological challenges in adapting and developing bioengineering technology for food production, including bioreactors, cell culture media, bioscaffolds, and cell lines, as well as major challenges in consumer acceptance and new food regulations [1]. Cultured meat will be composed of cells; therefore, ready access to reliable, safe, and culinarily appropriate sources of cells is required for both the research and eventual production of cultured meat [6]. A brief overview of the production of cultured meat is shown in Figure 1 for reference.

Although primary cell cultures may be used to study the mechanisms of cultured meat production over short time scales, primary cells can only undergo a finite number of cell divisions before they lapse into senescence or arrest of the cell cycle. This makes longer-term studies and commercial-scale production difficult. Using primary cells for cultured meat production requires that donor herds of animals be kept to provide biopsies, and that these biopsies are regularly acquired and approved for use in food production. Unlike primary cell cultures, immortal cell lines do not undergo senescence, and can exhibit infinite divisions. They could, therefore, be easier to study, and allow for safer and more consistent cultured meat without ongoing need for animal biopsies [7]. 

Currently, however, there are no cultured-meat-appropriate cell lines available to researchers and developers. The culinarily appropriate components of animal meat are primarily skeletal muscle and adipose tissue [3]; relevant cell lines to grow these tissues would be satellite cells and adipose-derived stem cells, mesenchymal stem cells, fibroblasts, and pluripotent stem cells from cows, pigs, chickens, turkeys, and seafood [8]. The closest existing cell lines are myoblasts from model species commonly used in research such as mice, rat, hamsters, and Japanese quail [9]. In addition to consumer perceptions of what animals are edible, existing cell lines would lack the taste, nutrition, and texture that consumers associate with meat, and have not been confirmed safe for consumption [10]. Past research has found that consumers expect cultured meat to taste worse than conventional meat, and that taste is essential for the long-term acceptance of cultured meat [11]. Taste, nutrition, and safety have been shown in consumer studies to be important concerns for the willingness to consume cultured meat [11,12]. Immortal cell lines used in cultured meat should therefore be developed from cell types and species familiar to consumers and that are tasty, nutritious, and food-safe.

Efforts have only recently begun in developing repositories of cell lines specifically appropriate for cultured meat in order to facilitate the research and development of novel food. One example is the partnership between the Good Food Institute (GFI) and Kerafast that aims to curate a repository of standardized terrestrial and aquatic cell lines appropriate for cultured meat research [11,13]. Thus far, only one cell line deposited at Kerafast has been identified as a candidate for cultured meat [6]

As no commercially available, agriculturally relevant, immortal cell lines have been confirmed food-safe and initial repositories are seeking deposits, this paper defines the existing methods to produce new cell lines and their limitations, and the unique considerations of cell lines for use in cellular agriculture.

## 2. Methods for Establishing Immortal Cell Lines

**Figure 2:**
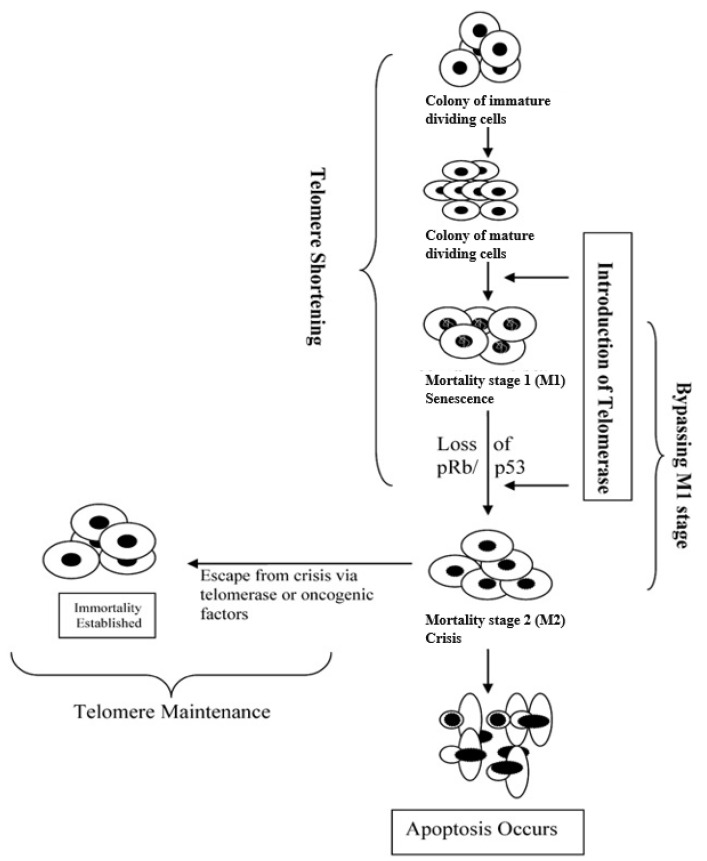
Immortalization can be induced by the expression of telomerase or factors inactivating or bypassing p53/p16/Rb. Reproduced with permission from Maqsood et al., Cell Biology International; published by Wiley Online Library, 2013.

Cell lines become immortal when they lose their cell cycle checkpoint pathways and circumvent the process of senescence. There are currently three methods to establish immortal cell lines: the discovery of spontaneously immortalized cell lines, expression of the catalytic subunit of telomerase (TERT), or induction by viral genes that inactivate p53/p14/Rb. Each method utilizes either telomerase expression or inactivation/bypassing of the cell cycle, or both, as summarized in Figure 2 [7]. These changes can occur naturally or be directed by genetic manipulation.

### 2.1. Spontaneous Immortalization of Cell Lines

In rare events, most often in cancer, cells will spontaneously immortalize. These cells can then be isolated. Tissue must be sampled by biopsy and then dissociated, and the cell types must be isolated and tested for proliferative capacity and identity. This was the original method to obtain cell lines beginning with the first immortal cell line derived in the 1940s from mouse fibroblasts, as well as the HeLa cell line isolated from the cervical cancer of Henrietta Lacks. Immortality of the HeLa cells could be attributed to the cells’ infection with human papillomavirus 18, which may have either degraded the tumor-suppressor protein p53 or caused chromothripsis, a chromosome-shattering and rearrangement associated with 2–3% of all cancers and which changes the expressions of thousands of protein-coding genes [14,15]. 

In addition to being discovered in samples, spontaneous immortalization can be coordinated by scientists. Cancer can be induced by radiation or chemical carcinogens. A cell line can also be put under serial passaging to select for clones with immortalization markers, high TERT expression or low p15/p16/Rb expression. One example of spontaneously immortalized cell lines in cultured meat is the chicken fibroblast line used by the company Future Meat. The Future Meat cell line was created by culturing fibroblasts isolated from a chick embryo, and isolating, concentrating, and expanding the foci of more rapidly growing cells until there was a culture of uniform morphology that was able to survive past the 20–30 divisions undergone by an unmodified somatic cell [16].

Spontaneous immortalization has its limitations, and may be more suitable for some situations and less suitable for others. For example, spontaneously immortalized cells would likely not be considered genetically modified (GM), which could allow them access to European markets that currently have strict regulations on GM foods [17]. Under other jurisdictions, however, spontaneously immortalized cells may be held with concern as equivalent to cancerous cells. As noted in the HeLa cell line, the process of spontaneous immortalization often results in a number of additional mutations that are not required for immortalization, and which may alter other aspects of the cells in unpredictable ways. Finally, different cell types have different predispositions towards spontaneous immortalization. Fish, for instance, have a high propensity for spontaneous immortalization due to the naturally high regenerative capacity of their adult stem cell population throughout their lifecycle [18], whereas mammals have more regulation checks in place to limit spontaneous immortalization [19,20].

### 2.2. Establishment by Telomerase

In a normal cell, chromosome telomeres shorten with every replication of DNA. Repeated shortening eventually exposes the chromosome ends to damage, which leads to cell senescence. The upper limit of divisions that a cell can reach before it enters senescence is known as the Hayflick Limit, and is typically between 20 and 30 divisions [21]. Germ line cells typically contain the enzyme telomerase, which counteracts telomere shortening in germ line cells, but this enzyme is absent in most somatic cells. The misexpression of telomerase in somatic cells can allow these cells to have infinite proliferative capacity [22]. 

The introduction of telomerase has been successful in immortalizing cell lines, because telomere elongation helps cells escape senescence triggered by telomere shortening. This has been performed by ectopic expression of the catalytically active subunit of telomerase (TERT), or by the overexpression of TERT. Despite the checkpoints controlling human cell immortalization [19], human fibroblast and keratinocyte cell lines have been immortalized by infection with retroviruses expressing human TERT [23,24]. Human endothelial cells have also been immortalized by the ectopic expression of hTERT via plasmid transfection [25]. The authors have not found any published work that applies this method to agriculturally relevant cell lines for cellular agriculture.

### 2.3. Establishment by Inactivation of the p53/p16/Rb Stress Response

**Figure 3:**
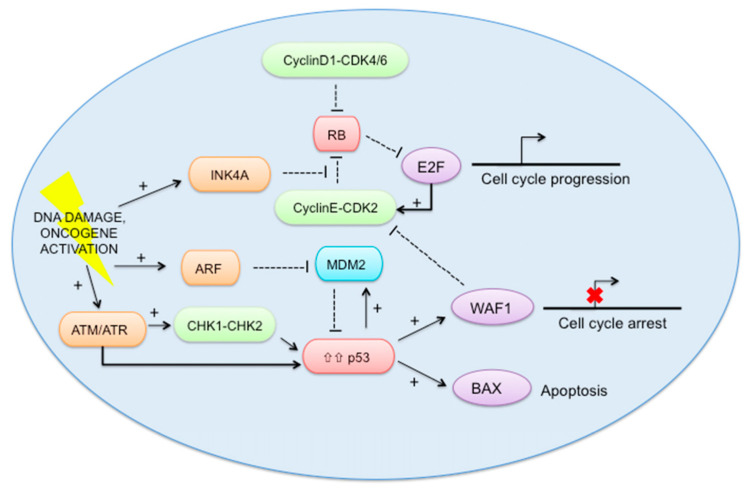
The pathways regulating cell cycle arrest [37]. Figure licensed under a CC BY 4.0 license.

Telomerase expression has not been as commonly successful in immortalization as methods that inactivate or bypass the p53/p16/Rb stress response (protein mechanisms shown in Figure 3). Activation of the transcription factor p53 is induced by DNA damage and other stress, causing cell cycle arrest until the cell determines that the DNA can be repaired. If the damage is deemed irreparable, p53 activates and triggers apoptosis and cell cycle arrest [26]. Activation of p16 and Rb stops other proteins from triggering DNA replication, resulting in cell senescence [26,27]. Inhibition or mutation of p16 and Rb, therefore, can allow cells to continue DNA replication, leading to cell division without regulation [7].

The earliest method for immortalizing cell lines via inactivation or bypass of the p53/p16/Rb stress response has been transformation with viral genes. A notable example is the simian virus 40 (SV40) large T-antigen (TAg), which binds to and inactivates p53/Rb as well as other tumor suppressor factors in a number of species and organ types [28,29,30]. In the wild, SV40 is thought to infect senescent kidney epithelial cells in Rhesus macaques; here, TAg reactivates the host cell in order to promote replication of the SV40 virion [31]. In most mammalian cells, however, the host can be transformed by TAg without viral assembly and cell death, leaving the host cell stably transformed for immortalization [32]. In addition to the SV40 T antigen, a number of other viruses and viral proteins, such as the E6 and E7 ORFs of human papillomavirus, the E1A and E1b proteins of adenovirus, and Epstein–Barr virus, have been used to induce cell line immortality by inactivating cell cycle checkpoints [33,34,35,36].

### 2.4. Combined Approaches to Establishment

In many cases, TERT expression or inactivation of p15/p16/Rb alone are not sufficient to immortalize a cell line, suggesting that both telomere shortening and the p53/p16/Rb stress response must be bypassed. Myoblasts have previously been shown to require the bypass of both senescence triggers to become immortal [38]. In 2016, the cultured meat company Upside Foods submitted a patent to immortalize cell lines by overexpressing TERT and using CRISPR to knock out expressions of p15 and p16 in skeletal chicken muscle cells [39]. The knockout of p15 and p16 alone increased the proliferative capacity of cells, but adding the overexpression of TERT by an ectopic TERT gene increased the capacity indefinitely. Other methods of immortalizing myogenic cell lines may bypass both telomere shortening and the p16 stress pathway by ectopically expressing TERT, and the Rb inhibitors cyclin-dependent kinase 4 (CDK4), and cyclin D1 (Figure 2) [40,41].

## 3. Unique Challenges for Cell Lines Used in Cellular Agriculture

### 3.1. Species-to-Species Variation in Establishment Difficulty 

Cellular agriculture requires cell lines made from organisms that are agriculturally relevant rather than well-characterized; therefore, some cell lines have been difficult to establish. In particular, no permanent cell line is available for marine invertebrates, such as mollusks or snails or crustaceans, perhaps due to a lack of information regarding their physiology and biochemistry and understanding of the variables required for their immortalization. To create these cell lines, scientists must first characterize their stem cell expression markers and ideal culture conditions, and must also develop methods for inducing myogenesis in the created cell lines [39]. Currently, the Mote Marine Lab is working to develop cell lines and supporting protocols for whiteleg shrimp, and are funding a project to characterize stem cell expression markers for abalone and oysters [42]. Even for species such as fish that spontaneously immortalize frequently compared to other animals, there is often a lack of knowledge of molecular and genetic markers, and very few species-specific antibodies available to aid identifying appropriate cells [18]. 

### 3.2. The Food Safety of Cell Lines

One of the most common concerns about cultured meat from consumers is the safety of ingesting cell lines. Currently, cultured animal cell lines are not commonly eaten by consumers. An immortal cultured meat cell line may contain expressed oncogenes, and if so, food products produced with these cells will need to be confirmed not to have tumorigenicity [43,44]. Although there is limited evidence to suggest that DNA from genetically engineered plant cells can integrate or be transferred into somatic cells or the microflora of the human gastrointestinal tract [45], due diligence would require further investigation in genetically engineered animal cells. Confirming that future products made from immortalized animal cells expressing oncogenes, either through spontaneous immortalization or genetic engineering, would be safe represents a gap in knowledge in this field. In addition, future immortalized cells should also undergo physico-chemical inspection throughout the production process, and ultimately be tested for safe consumption. Cells expressing novel levels of non-native compounds, such as enhancement with carotenoids, will require special confirmation that these levels are safe for human consumption. Oncogene expression could be tested during the manufacturing process of cultured meat by sampling a patch from a small portion of the cells which should be an accurate representation of the entire population in a cell line [44,46]. 

In the case that a cell line is confirmed safe for consumption, it will still need to be regularly monitored for contamination and genetic drift. Contamination of cell lines with other cell lines and adventitious agents can be common in cell culture [44,46,47]. Animal-sourced components of cell culture media, such as fetal bovine serum, are frequently at risk of harboring adventitious agents [44]. In part because of these challenges (along with other issues such as high cost, limited availability, and animal welfare concerns), non-animal-origin reagents are being extensively studied for cultured meat [44]. Genetic drift is also a known occurrence in cell lines where, over time, mutations build up that eventually cause changes in phenotypes [48]. To mitigate the risk of losing cell line fidelity to genetic drift, cell banks must be built in which cryopreserved cultures of relevant cell lines are quality-controlled and protected against the presence of viruses, bacteria, yeast, and mycoplasma. Such banks may include both a master cell bank, a collection of cells of uniform composition derived from a single source, and a working cell bank, a collection of cells derived from one or more vials of cells from the master bank expanded by serial subculture for use in cultivation. To create a cell bank, cells will need to be selected, validated, and cryopreserved in small batches that can later be thawed, validated again, and expanded for cultivation [44]. Cryopreservation techniques should be used that are animal-free and confirmed safe for cultured meat. Banked cells will be stored in multiple locations at ultra-low temperatures, likely under liquid nitrogen. During storage, there may be the potential for contamination by liquid nitrogen transferring pathogens to cells or cross contamination due to the leakage of cryopreservation bags. Storage in the vapor phase rather than the liquid phase might reduce the potential for otherwise possible cross-contamination, because liquid nitrogen has the potential to transfer pathogens to cells, even if stored in freezing bags [44,49]. Cell line authentication and screening will be critical in controlling contamination [44]. Cultures can be compared to these preserved cell lines to ensure quality control and replaced with serial subcultures of the preservation vials [44,48]. Similar cell banks exist for vaccination cultures [50]. 

Regulatory agencies are beginning to adapt pathways for cultured meat products to be examined regarding safety for consumption. To some degree, this will involve updating the existing regulations on cell-derived products for relevance to consumption as food [51,52,53]. Although animal-cell-cultured food has not been marketed before, cell lines have been used previously to create food ingredients or additives, such as enzymes, oils, and transgenic proteins, and also for numerous cell therapies [51]. For cultured meat, the Singapore Food Agency (SFA) became the first national agency to approve a cultured meat product, a cultured chicken nugget made with fetal bovine serum, in December 2020 [54]. To receive approval, the chicken was reviewed by the SFA via a “novel food” petition, which included a description of the cultivation process, the nutritional composition and characterization of the final product, information related to cell lines, scaffolding, media, and safety assessments covering possible hazards as well as other relevant safety studies such as digestibility assays and allergenicity profiling [55]. The petition’s specific information on cell lines entailed a description of the cell line source, description of the modifications and how these relate to the expression of substances that may result in food safety risk, description of the methods used for selection, screening, preparation, and banking, and information on how the purity and genetic stability of cell culture is ensured during the manufacturing process [56]. In the United States, the Food and Drug Administration (FDA) has announced that they will conduct pre-market consultations with companies on a product basis to evaluate the safety of cell lines, as well as production materials/process, tissue collection, components, and inputs [57]. The consultations involving cell lines will include collaborations with the Food Safety and Inspection Service (FSIS, an agency of the United States Department of Agriculture), which will regulate the product after cell harvest. After products are marketed, the FDA will oversee initial cell collection, the development and maintenance of qualified cell banks, proliferation, and the differentiation of cells through time of harvest [56]. In the European Union, cell-cultured meat may be regulated under the Novel Foods Regulation, although cultured meat from engineered immortalized cell lines may be regulated under separate approval under Regulation No. 1829/2003 regulating foods containing or produced from GM organisms [57,58]. 

Regulatory bodies in a few other regions have begun to adapt their regulations to new cultured meat products [57].

### 3.3. Cell Lines and the Consumer Acceptance of Cultured Meat

The immortalization method itself may be an important factor in the decision of consumers to accept cultured meat. To the best of the authors’ knowledge, no study has yet compared the consumer acceptance of cultured meat made of primary cells and cultured meat made of immortalized cells. There have, however, been initial studies analyzing the acceptance of genetically modified and non-genetically modified cultured meat. These studies found that consumers are more willing to purchase non-genetically modified cultured meat compared to genetically modified cultured meat [59,60]. Products with genetic modifications for immortalization may therefore have lower consumer acceptance compared to similar products without genetic modifications, such as products made with primary cells, spontaneously immortalized cells, or cells immortalized by a footprint-free genetic engineering method with any gene manipulations removed from the final product. Consumer acceptance of food biotechnology has been found to be negatively affected by perceived unnaturalness (“tampering with nature” due to biological transformations), neophobia (fear of novel foods), and social distrust of the food industry [61]. For cultured meat in particular, distrust and the perceptions that it is unnatural or not real meat have been shown to be strong drivers in consumers’ willingness to eat cultured meat compared to the perceived benefits [11,61,62,63]. 

There are, however, some differences across applications and among consumers in how genetic engineering is evaluated that may inform decisions on the immortalization of cell lines for cultured meat. Consumers appear to be more concerned when genes are exchanged between different species, and see gene insertion as more unnatural than gene deletion [64,65,66]. Unless genetic engineering has tangible benefits for consumers (for example, cheaper, better tasting, nutritionally enhanced food), consumers’ negative perception of genetic modifications may have a compounding effect in lowering the acceptance of genetically modified cultured meat [67,68]. 

As mentioned in Section 1, the cell types and species that consumers may accept most readily for consumption differ from the type and species used in other cell line applications. Other cell line applications for myoblasts, for instance, use the myoblasts of model organisms such as mice, rat, hamsters, and Japanese quail [9], whereas these species are not typically eaten by consumers in many food cultures. Rather, the most commonly consumed animals globally are poultry, pigs, and cattle [68]. Food neophobia appears to be a major factor in many consumers’ acceptance of cultured meat, suggesting that cultured meat could find less acceptance if made from species and cell types that are unfamiliar to consumers [11,62,69,70,71,72,73]. Consumers’ preferred cell type and the extent of food neophobia will also differ across food cultures. Chinese and American consumers have been found to have lower food neophobia compared to Indian consumers and are accustomed to a more diverse array of meat [69]. As an example of preferred cell type, fish maw is relatively expensive and desirable in Chinese markets, but unfamiliar to Western consumers, making it a potentially more desirable target for cultured meat in China than in the West [73]. 

Furthermore, some cell lines may be prohibited from consumption under religious food restrictions. Jewish kosher laws and Islamic halal laws do not allow the consumption of conventional pork and other meats [74], and it is still be debated as to whether these laws may be similarly interpreted to not allow the consumption of cultured meat derived from the same species. These religious restrictions depend not only on species, but on the method of meat production [75]. Many Hindus do not eat conventional meat because they consider it against their principle of nonviolence, so they may not accept cultured meat produced with harm to animals [76]. Some rabbis and Islamic jurists have expressed that their rulings on cultured meat will depend on whether the cells are from a kosher-slaughtered animal or a halal-slaughtered animal, respectively, and whether production includes use of blood or serum [75,76,77,78,79]. 

### 3.4. Other Unique Attributes to Select for in Cultured Meat Cell Lines

Cell lines for cultured meat production will not only be from animals and cell types not previously established, but will also require different attributes from those previously typical for immortal cell lines. Specifically, they should be food-safe, able to proliferate stably and efficiently in a large-scale production environment with minimum costs, and have desirable taste, texture, and nutrition. 

Multiple companies have filed patents on ways to overcome the unique challenges of growing food-relevant cells at scale. The cultured meat company Wild Type has filed one patent on a footprint-free method of genetic modifications to control cell differentiation and proliferation with a small genetic footprint [80]. Another company, Upside Foods, has filed one patent to genetically engineer their pig cell line O2K so that they can replace some growth factors with small molecules, reducing the need for expensive animal serum or supplemental biologics in the high volume of media required for cell production [81,82]. The same company has also filed a patent to genetically modify cell lines with an overexpression of glutamine synthetase to convert ammonia into an amino acid [83]. Ammonia build-up is a challenge for large-scale cell culture, because it has known inhibitory and toxic properties to cell culture and bioreactors cannot replicate the uptake of ammonia by the bloodstream as in a complete organism [84]. Overexpression of glutamine synthetase could also provide an additional amino acid source for cells [83]. Modifications such as these aim to make cultured meat cell lines safer, less expensive, and more efficient to culture at large scales [58,59,60].

The sensory experience of cultured meat may be affected by the cell types used. A scientific paper on the sensorial qualities of cultured meat compared to animal-sourced meat has not been published thus far [85]. Although other inputs such as feed or media may likely affect the sensorial qualities of cultured meat, future research may indicate innate differences in taste and texture between cultured cell types [85,86]. The inclusion of cell types commonly found in conventional meat, including myofibers, fibroblasts, adipocytes, endothelial cells, and ECM-producing supporting cells, could improve the ability of cultured meat to produce similar tastes and textures to conventional meat [8,85]. In order to produce cultured meat with the mentioned cell types, cultured meat cell lines would either need to be made from these cell types or from satellite cells and adipose-derived stem cells, mesenchymal stem cells, or pluripotent stem cells able to be differentiated in vitro into these cell types [8,87]

The sensory experience of cultured meat may additionally be affected by certain attributes to be selected for in cell lines. Consumer surveys have found that many consumers expect cultured meat to be less tasty than conventional meat, and this perception contributes to their unwillingness to consume cultured meat [11,12]. The sensory experience of meat generally involves taste and aroma from the Maillard reaction and lipid oxidation reactions, color from heme proteins, and a tender, juicy texture [85]. Techniques have begun to be studied in modifying the nutritional profile of cultured meat cell lines, and could also be applied to alter the taste and aroma of cultured meat by variation in the production of different flavor compounds within cells [88]. Cultured meat’s color is generally paler than conventional meat because the expression of the heme protein myoglobin is suppressed at ambient oxygen conditions. In order to recreate the color of conventional meat, myoglobin or another colorant could be directly added to cultured cells, cultivation could be adapted to low oxygen conditions, or cell lines could be engineered to express myoglobin at the oxygen level of cultivation [85,89]. Cultured meat’s texture would differ from that of structured meat unless adipose and muscle cells were able to be co-cultured together, either on a scaffold mimicking connective tissue or on an extracellular matrix made by the cells themselves [85]. In addition to the intrinsic taste of each cell, the ability of adipose and muscle cell lines to be co-cultured into thick tissue may become crucial for structured cultured meat products. Fat contributes to meat’s taste, aroma, juiciness, and tenderness, and successful co-culturing could create structured meats such as steaks or pork chops [90]. Thick tissue with strong binding is used for a number of meat products, and would require the use of external binders if cells are not able to create these structures themselves in vitro [85]. All these cell attributes together could affect the sensory experience of cultured meat [85,90]. Although no data are publicly available, several groups have announced plans to work with cells from specific heritage breeds, with the idea that genetics partially determines the sensory properties of meat [91,92,93]: Cell Farm Food Tech aimed to produce mesenchymal stem cell lines from their native Argentinian cattle breeds [94], and three groups are researching the use of cells from Wagyu cows to make cultured beef [87,95,96]. Further evaluations of how a donor animal’s species, age, gender, and provenance affect the taste and texture of cultured meat produced with its cells could help ensure that cultured meat is not only safe, but desirable to consumers [85,97]. 

Cultured meat should be designed to provide similar, if not enhanced, available levels of nutrients compared to conventional meat. Meat provides 26% of the global protein supply, 24% of the global fat supply, and 9% of the global calorie supply per capita per day, as well as essential micronutrients such as iron, zinc, and vitamins A and B12 [98]. For cultured meat to replicate the dietary place of meat, it must provide similar levels of these nutrients through some combination of components including cells, scaffolding, and added nutrients. The combination of these components will contribute to the nutritional composition as well as the bioavailability of these nutrients, because the ability to digest, absorb, and metabolize nutrients is affected by the matrix in which they are incorporated. Cell line attributes relevant for nutrition can therefore be categorized as the following: their ability to co-culture with other cell types to achieve a nutritional composition similar to conventional meat, their ability to take up nutrients added to its media to supplement the nutrition of the end product, their specific percentage content of fat and protein, their fatty acid and amino acid composition, and the bioavailability of their nutrients depending on the matrix within which it is consumed [75,85]. Some important nutrients in conventional meat, including essential fatty acids and vitamin B12 and minerals, are not produced in muscle cells but are derived from animal feed components which have been digested and modified by non-muscle organs. Unless specifically added to the culture medium and taken up by the cells, or added in post-harvest processing (Figure 1), these compounds would be absent from cultured meat, influencing nutrition [85].

Other cultured meats could be engineered for desired differences from the nutritional profile of conventional meat. As an initial exploration of nutritional engineering for cultured meat, Stout et al. have incorporated a biosynthetic pathway for carotenoids, a class of antioxidants native to some plants but not to animals, into primary bovine satellite cells (BSCs) [88]. They demonstrated that these carotenoid-producing BSCs showed antioxidant capacity and reduced lipid oxidation, potentially increasing the nutritional value of meat and reducing the link between red meat consumption and colorectal cancer [88]. There is a wealth of possible research in tuning the nutritional characteristics of cultured meat, even including edible therapeutics [88].

## 4. Conclusions

Although there are several techniques used to establish cell lines appropriate for bio-logical or medical research, there are still several unanswered questions about how to establish immortalized cell lines suitable for human consumption as cultured meat and which of these existing techniques are appropriate or would need to be modified for use in cultured meat. The lack of readily available cell lines is a current barrier to conducting research in cultured meat. Most academic cultured meat research, from media composition to bioscaffolding to bioreactor projects, must currently begin by creating cell lines for study. 

Cultured meat will require unique cell lines that are safe, appropriate for industrial-scale production, and desirable to consumers. Although development of these cell lines can be based on established immortalization methods, they will need to be applied to cell types appropriate to meat. In addition, the properties of food safety, industrial production, and consumer desirability have only begun to be explored in cell lines for cultured meat. There is still much work to be done before a collection of cell lines appropriate to cultured meat can be established and readily available for the use of researchers and developers. 

## Data Availability

Not applicable.
